# The International Medical Congress Again

**Published:** 1886-01

**Authors:** 


### THE INTERNATIONAL MEDICAL CONGRESS AGAIN.

In our last number was published the report of the conference of dentists in Buffalo, called to consider the propriety of completing the organization of a Dental Section in the International Medical Congress, of 1887. Some of those present traveled nearly two thousand miles to attend it. The meeting unanimously voted that it was inexpedient, under the present circumstances, to establish such a Section.

At the banquet of the late meeting of the First District Dental Society, in New York, where representative dentists were present from nearly all sections of the country, an expression of opinion was obtained by vote. No speeches to influence the minds of those present were made, but it was requested that all who believed it inexpedient that a Dental Section should be established should rise. All but two stood upon their feet at once.

In view of these most unequivocal expressions of opinion upon the part of those who are best acquainted with the status of affairs, it seems to us that the duty of such as were appointed to official positions in the Section, or who may be offered such, is plain. A Section cannot be organized without dividing dentistry as medicine is now divided. It matters not what the right of the matter is, nor which of the opposing medical factions is most in fault. The existing fact is that only a portion of the medical profession will take any part in the Congress, and that portion, neither in point of numbers nor influence, can claim to be the great body of the profession. A successful Congress cannot be organized without unity, and the breach is so wide that it now seems impossible that it should be bridged over. It is idle to assert that the differences are upon the point of settlement. It is no mere insignificant faction of malcontents who decline to have anything to do with the Congress. It comprises some of the best men in the profession. We might say that it is composed of *the* best men of medicine. They are not factional in spirit, and will not openly oppose the Congress, but they will have nothing to do with it, and without their aid foreign delegates cannot be induced to attend.

This is the exact condition of affairs to-day. What has brought this about is not unknown to the readers of this journal. Let the blame rest where it may, the absolute truth is that the Congress will, in the main, be one of second and third rate men, and with such an one dentists should have nothing to do. How can we invite foreign dentists to attend a meeting such as this promises to be? What would be our feelings in the face of the invidious comparisons that must be made between this and the London meeting? With what propriety could we ask the establishment of a Dental Section in future Congresses, when it was known that we were compromised by taking part in a factional Congress? It seems to us that the part of wisdom is for us to stand aloof for the present. If, when the American Medical Association shall meet next spring, it shall recede from the unwarranted position that the Congress shall be dominated by that or any other single society, then we can all join in and act together. At present such a thing is impossible.

Of those who were appointed officers of the Section, when it was first organized under happier auspices, all but four have resigned, and of these four we know of but two who have definitely accepted and will continue to act. From the other two word has not been received. A number of additional members of the Council have been appointed, and a number of these have declined. Does this look like harmony among ourselves? Does it promise even a partial success, when so great a majority of those who were invited to prominent positions in the Section decline? For the sake of peace and harmony among ourselves, and because we believe that the best interests of dentistry demand it, we earnestly hope that the full organization of the Section will not at this time be pushed. Let us follow the example of the American Ophthalmological Society, and stand aloof, unless we can go in unitedly.

Let it be understood that we are not canvassing the merits of the medical quarrel at all. We are simply looking at existing, undisputable facts. The dentists should consider the matter as it is, and not according to what it might, could, would, or should have been. The simple truth is that only a comparatively small proportion of the profession will take part in the Congress, unless there shall be some radical changes made, and it is not advisable that dentistry be ruptured in twain, even though it were possible for a successful meeting to be held.

